# Current News

**Published:** 1893-12

**Authors:** 


### Current News.

### THE UNIVERSITY OF PENNSYLVANIA.

All the Departments of this institution have now been arranged upon a similar basis as regards length of course. The extension of the Department of Dentistry to eight months brings them all into harmonious working order. Beginning with the present session, the winter sessions of each will commence October 1, and close the first week in June.

### RECENT PATENTS.

Following is a list of recent patents, reported specially for the International Dental Journal:

500,139.—Dental Engine. Roswell De L. King, New York, N. Y. Filed September 24, 1892.

501,075.—Dental Engine. William A. Johnston and Arthur W. Browne, Prince's Bay, N. Y., assignors to the S. S. White Dental Manufacturing Company, Philadelphia, Pa. Filed March 16, 1893.

501,127.—Dental Tool. Henry C. Wofford, Greenville, S. C. Filed October 27, 1892.

501,429.—Artificial Tooth. Max J. F. Kneiff, Berlin, Germany. Filed August 2, 1892.

501,741.—Dental Articulator. George W. Simpson, Santa Barbara, Cal. Filed September 22, 1892.

502,164.—Dental Articulation-Cup. George K. Bagby, New Berne, N. C. Filed November 26, 1892.

502,209.—Apparatus for casting Aluminum Dental Plates. Warren M. Sharp, Binghamton, N. Y. Filed February 10, 1893.

502,352.—Dental Plugger. Alonzo A. Dillehay, Meridian, Miss. Filed June 7, 1892.

503,258.—Dental Disk and Carrier. Rufus G. Stanbrough, New York, N. Y. Filed January 29, 1892. Renewed January 18, 1893.

503,419.—Preparing Dental Fillings. Albert W. Johnston, New Brighton, N. Y., assignor to the S. S. White Dental Manufacturing Company, Philadelphia, Pa. Filed January 16, 1893.

503,737.—Dental Engine. Arthur W. Browne, Prince's Bay, N. Y., assignor to the S. S. White Dental Manufacturing Company, Philadelphia, Pa. Filed April 29, 1893.

503,740.—Dental Engine. Constant Doriot, Philadelphia, Pa., assignor to the S. S. White Dental Manufacturing Company, same place. Filed March 2, 1893.

503,744.—Dental Drill. Woodbury S. How, Philadelphia, Pa., assignor to the S. S. White Dental Manufacturing Company, same place. Filed June 26, 1893.

503,826.—Artificial-Tooth Mould. Robert Brewster, New Barnet, England. Filed April 17, 1893.

504,126.—Artificial Tooth. Charles C. Dure, Plymouth, Ind. Filed April 18, 1893.

504,487.—Dental Engine. Benoni S. Brown, Hyde Park, Mass., assignor to the S. S. White Dental Manufacturing Company, Philadelphia, Pa. Filed March 26, 1890.

504,489.—Dental Plugger. Constant Doriot, Philadelphia, Pa., assignor to the S. S. White Dental Manufacturing Company, same place. Filed March 9, 1893.

504,490.—Dental Engine. Constant Doriot, Philadelphia, Pa., assignor to the S. S. White Dental Manufacturing Company, same place. Filed March 11, 1893.

504,491.—Dental Engine. Constant Doriot, Philadelphia, Pa., assignor to the S. S. White Dental Manufacturing Company, same place. Filed March 13, 1893.

505,121.—Dental Disk-Holder. Edward Nelson, Frederick, Md. Filed January 11, 1893.

505,490.—Dental Disk-Holder. Rufus G. Stanbrough, New York, N. Y. Filed April 4, 1892.

506,200.—Dental Chair. George W. Archer, Rochester, N. Y., assignor to the Archer Manfacturing Company, same place. Filed October 7, 1892.

506,350.—Dental Disk-Holder. William H. Towne, Worcester, Mass., assignor to the S. S. White Dental Manufacturing Company, Philadelphia, Pa. Filed June 26, 1893.

506,522.—Dental Apparatus for grinding Teeth. Daniel E. Morse, New York, N. Y. Filed August 15, 1893.

506,762.—Process of securing Dental Suction-Valves to Plates. Alfred E. Ahrens, Stratford, Canada. Filed May 18, 1893.

Reissue.—11,376.—Dental Engine. John S. Campbell, London, England, assignor to Edward A. Pierce, New York, J. Otis Cox, Brooklyn, and the Carroll Aluminum Manufacturing Company, New York, N. Y. Filed December 17, 1889. Original No., 397,169, dated February 5, 1889. Patented in England March 26, 1888, and in France May 31, 1888.

Designs.—22,668.—Dental Clasp. John Kerr, Detroit, Mich., assignor to the Detroit Dental Manufacturing Company, same place. Filed June 5, 1893. Term of patent 7 years.

Trade-Marks.—23,276.—Dental Dams. I. B. Kleinert Rubber Company, New York, N. Y. Filed May 20, 1893. Essential feature, the word "Kleinert" on the representation of a Maltese cross.

23,402.—Anæsthetics. Charles E. Hale, Boston, Mass. Filed June 14, 1893. Essential feature, the words "Hale Method."

23,486.—Dentifrice. S. C. Wells & Co., Le Roy, N. Y. Filed June 9, 1893. Essential feature, the shaded representation of a scalloped shell, and a short word printed thereon.

23,558.—Tooth-Wash. James W. Johnson, New York, N. Y. Filed June 29, 1893. Essential feature, the word "Lavodent."

23,723.—Tooth-Paste. Joseph Spyer, Chicago, Ill. Filed July 21, 1893. Essential feature, the word "Mexican" arranged within a circle.

